# Dental Pulp Cell Transplantation Combined with Regenerative Endodontic Procedures Promotes Dentin Matrix Formation in Mature Mouse Molars

**DOI:** 10.3390/cells13040348

**Published:** 2024-02-16

**Authors:** Jorge Luis Montenegro Raudales, Yuta Okuwa, Masaki Honda

**Affiliations:** Department of Oral Anatomy, School of Dentistry, Aichi Gakuin University, 1-100 Kusumoto-cho, Chikusa-ku, Nagoya 464-8650, Aichi, Japan; ohagighost@yahoo.co.jp (Y.O.); honda-m@dpc.agu.ac.jp (M.H.)

**Keywords:** regenerative endodontics, dentin–pulp complex, odontoblasts

## Abstract

Regenerative endodontic procedures (REPs) are promising for dental pulp tissue regeneration; however, their application in permanent teeth remains challenging. We assessed the potential combination of an REP and local dental pulp cell (DPC) transplantation in the mature molars of C57BL/6 mice with (REP + DPC group) or without (REP group) transplantation of DPCs from green fluorescent protein (GFP) transgenic mice. After 4 weeks, the regenerated tissue was evaluated by micro-computed tomography and histological analyses to detect odontoblasts, vasculogenesis, and neurogenesis. DPCs were assessed for mesenchymal and pluripotency markers. Four weeks after the REP, the molars showed no signs of periapical lesions, and both the REP and REP + DPC groups exhibited a pulp-like tissue composed of a cellular matrix with vessels surrounded by an eosin-stained acellular matrix that resembled hard tissue. However, the REP + DPC group had a broader cellular matrix and uniquely contained odontoblast-like cells co-expressing GFP. Vasculogenesis and neurogenesis were detected in both groups, with the former being more prominent in the REP + DPC group. Overall, the REP was achieved in mature mouse molars and DPC transplantation improved the outcomes by inducing the formation of odontoblast-like cells and greater vasculogenesis.

## 1. Introduction

Complete regeneration of the teeth remains a significant clinical challenge because of the unique structure of the dentin–pulp complex, which is composed of innervated and vascularized connective tissue with specialized cells called odontoblasts that extend their projections to the rigid walls of the dentin enclosing the structure. These components function in conjunction with intricate dynamics to maintain tissue homeostasis in the pulp [1,2]. In immature teeth, root formation is halted when severe injury occurs to the pulp, either in the form of trauma or caries. In these cases, the most widely used treatment is apexification, which allows for the formation of a calcified barrier at the apical region before commencing conventional root canal treatment; however, this treatment is limited in that it does not induce root development and thus the teeth are still prone to root fracture [3,4]. Therefore, a regenerative endodontic procedure (REP) has been developed as a biological-based alternative to regenerate authentic dentin–pulp components and promote root formation. REPs are defined as “biologically based procedures designed to replace damaged structures, including dentin and root structures, as well as cells of the pulp–dentin complex.” [5]. In contrast to conventional root canal treatment, REP consists of disinfecting the canals followed by over-instrumentation to induce bleeding, creating a blood clot that allows for the influx of multipotent mesenchymal stromal cells (MSCs) to promote new tissue formation [6,7,8]. In a recent systematic review, Li et al. [9] determined that the success rate of REPs in immature teeth was 95.6%, in which “success” was defined as the loss of any symptoms and disappearance of the periapical lesion. However, data from another recent systematic review of radiographic and histological outcomes of REPs in 14 immature permanent teeth showed that although root maturation with root lengthening had occurred signs of histological success remained elusive, and most of the regenerated tissues consisted of vascularized, innervated fibrous tissue with deposits of cementum- or bone-like tissue and the absence of odontoblast-like cells in most cases [10]. In addition to the importance of reducing symptomatology in patients, proper regeneration of the teeth would ideally lead to even better outcomes such as reduction in the risk of root fractures [11], thus prolonging the lifespan of the treated teeth in the mouth. Overall, these findings highlight the enormous potential of REPs as well as the pitfalls that must be overcome to achieve desired results.

Owing to the clinical effectiveness of REPs in immature teeth, these procedures have also been utilized in mature human teeth to regain immune system functionality and sensitivity, which is not possible with conventional root canal treatment [11,12]. However, achieving REP in the mature teeth remains a challenge, owing to anatomical differences such as a closed root apex and a decline in the proliferation and migration ability of endogenous stem cells in the vicinity of the tooth [13,14]. Therefore, MSC transplantation can offer an exogenous source of progenitor cells. MSCs have been widely studied in regenerative medicine because of their multilineage differentiation and proliferative abilities [15,16,17]. In addition, MSCs exert regenerative effects through immunomodulation, recruitment of endogenous progenitor cells, and paracrine effects through their secretome, enabling the transfer of nucleic acids and proteins [18]. MSCs can be isolated from several tissues, and in particular, various types of MSCs can be found in the relatively small area of tissues in the oral region. These MSCs include stem cells from human exfoliated deciduous teeth (SHEDs) [19], dental follicle stem cells (DFSCs) [20], stem cells from apical papilla (SCAPs) [21], and periodontal ligament stem cells (PDLSCs) [22]. In addition, dental pulp cells (DPCs) include endothelial cells, neurons, fibroblasts, odontoblasts, and dental pulp stem cells (DPSCs), which are a distinct population of MSCs that are well known for their differentiation into mesenchymal lineages such as osteoblasts and adipocytes as well as non-mesenchymal lineages such as endothelial cells [15,23]. Several reports document the important functions of DPCs. Han et al. reported that DPCs produce high levels of molecules that induce cell proliferation such as hepatocyte growth factor (HGF) [24], while others have reported the immunomodulatory function of DPCs through the suppression of lymphocyte proliferation and promotion of an anti-inflammatory response [25,26]. Due to their great differentiation potential, DPCs have been used in studies for neuronal [27,28], periodontal [29], and osteogenic regeneration [30]. DPCs can differentiate into dentin-secreting odontoblast-like cells both in vitro and in vivo [31,32]. Furthermore, they can be obtained relatively easily compared to other sources of MSCs, and their low immunogenicity shows potential for allogeneic transplantation [33,34]. Overall, these advantages make DPCs ideal cell-based candidates for regeneration of the dentin–pulp complex in mature teeth.

Although there have been some preclinical and clinical studies on the local transplantation of DPSCs into teeth, the local transplantation was performed after conventional root canal treatment and the teeth used in the clinical studies were from young patients (7–12 years old) [34,35]. Therefore, information regarding the effects of DPC transplantation in mature teeth is scarce, as current strategies to evaluate the success of REP treatment in patients rely on radiographic evidence of the disappearance of periapical lesions and a positive response to sensitivity tests owing to practical considerations for performing histological analyses in mature teeth [10,11]. This lack of histological analysis has hindered progress in the field without sufficient evidence to establish conclusions regarding the optimal strategies to approach REP in mature teeth. Therefore, the use of animal models is of paramount importance in this regard. Small-animal models such as mice are convenient because they are easy to handle, available in great numbers, and inexpensive compared to larger animals. In addition, murine pulp inflammation models have already been established, and it is more feasible to create transgenic lines in mice than in other species [36]. Accordingly, using mouse models for REPs will accelerate treatment development by allowing researchers more freedom in experimental design and generating large volumes of histological data.

To the best of our knowledge, there are no reports of the local transplantation of DPCs in mature mouse molars after inducing bleeding during conventional REP treatment. In immature teeth, the blood clot alone has been shown to be equally effective as a scaffold and promoter of tissue regeneration when compared to the scaffold using plasma rich in growth factors (PRGF). [37]. Murine DPCs, like human, have neural crest origins with the capacity to differentiate into dentin-producing odontoblast-like cells [38], a unique and chief component of the dentin–pulp complex [39]; thus, we focused on assessing murine DPCs as a source for allogenic cell transplantation. Therefore, we hypothesized that the blood from REPs serves as a scaffold to provide the appropriate nutrients and stability for the survival of transplanted cells. To test this possibility, the aim of this study was to explore the effects of DPC transplantation combined with REPs on mature mouse teeth based on histological evidence, including the differentiation of dentin-producing odontoblast-like cells and improved vasculogenesis.

## 2. Materials and Methods

### 2.1. Animals

The study protocol was approved by the Animal Care and Use Committee of the School of Dentistry, Aichi Gakuin University (approval no. AGUD495). All animal handling and surgical procedures were performed in accordance with the Regulations on Animal Experimentation of the School of Dentistry at Aichi Gakuin University. Forty-six 5-day-old C57BL/6-transgenic CAG-enhanced green fluorescent protein (EGFP) mice and 46 C57BL/6NCr mice (10-week-old) (Japan SLC, Shizuoka, Japan) were used in this study.

### 2.2. Isolation and Culture of Transgenic Mouse DPCs

DPCs were isolated and cultured as described previously [38,40]. Briefly, molars from 5-day-old C57BL/6-transgenic CAG-enhanced green fluorescent protein (EGFP) mice (Japan SLC, Shizuoka, Japan) were extracted from dissected hemimandibles. The pulp tissue was isolated and submitted to enzymatic digestion at 37 °C for 1 h with 1.2 units/mL dispase II (Roche, Basel, Switzerland), 2 mg/mL of collagenase type IV (Worthington Biochemical Corporation, Lakewood, NJ, USA), and 2 mM CaCl_2_. After diluting the digested product with stem cell medium (60% low-glucose Dulbecco’s modified Eagle medium (Gibco, Invitrogen, Carlsbad, CA, USA), 40% MCDB201 (Sigma, St. Louis, MO, USA), 2% fetal bovine serum (Gibco, Invitrogen), 1X Insulin-Transferrin-Selenium (Sigma), 1:100 linoleic acid-albumin derived from bovine serum albumin (BSA; Sigma), 10^−9^ M dexamethasone (Sigma), 10^−4^ M ascorbic acid 2-phosphate (Sigma), 1 × 10^3^ units/mL leukemia-inhibitory factor (LIF-ESGRO, Millipore), 10 ng/mL epidermal growth factor (Sigma), 10 ng/mL platelet-derived growth factor subunit B (R&D Systems, Minneapolis, MN, USA), and 10,000 U/mL 1× penicillin-streptomycin (Gibco, Invitrogen) [38,40,41], the cells were filtered with a 70-µm nylon strainer (BD Falcon, Corning, NY, USA) and centrifuged at 300× *g* for 10 min at room temperature. The pellet was dissolved, and cells were plated in 6-well plates in the stem cell medium. The medium was replaced the following day and every other day, and the cells were split at a 1:4 ratio for subsequent experiments.

### 2.3. Immunocytochemistry of DPCs

DPCs were cultured in cell culture slides (Falcon^®^ CultureSlides, Corning, NY, USA) with stem cell medium at a density of 7 × 10^3^ cells/chamber. The DPCs were washed with phosphate-buffered saline (PBS) and fixed with precooled methanol for 10 min. The cells were treated for 1 h at room temperature with 10% goat serum and 10% donkey serum to block non-specific binding. The cells were then incubated with anti-vimentin at 10 µg/mL (MAB2105, R&D Systems) and anti-wide-spectrum cytokeratin (1:75, ab9377, Abcam, Cambridge, UK) overnight at 4 °C. For the negative controls, the primary antibodies were omitted. After two washes with PBS for 5 min each, DPCs were incubated for 1 h at room temperature with the secondary antibodies donkey anti-rat Alexa Fluor^®^ 647 (ab150155, Abcam) and Alexa Fluor^®^ 594 goat anti-rabbit (a11012, Life Technologies (Carlsbad, CA, USA), both at 1:200 dilutions. Coverslips were mounted with Vectashield mounting media containing DAPI (Vector Laboratories, Newark, CA, USA). Slides were visualized using a Keyence BZ-X710 microscope (Keyence Corporation, Osaka, Japan) and the number of vimentin+, vimentin+/cytokeratin+, and cytokeratin+ cells, with stained cytoplasm were quantified from 10 fields of vision with a 20× objective lens. For KLF4 staining, DPCs at the second, fourth, and fifth passages were cultured and fixed as described above and then permeabilized with 0.1% Triton-X and 1% BSA for 10 min. Endogenous peroxidase activity was blocked with 0.3% hydrogen peroxide (FUJIFILM Wako, Osaka, Japan) for 30 min and then the cells were treated with 10% goat serum to inhibit non-specific binding. The cells were incubated overnight at 4 °C with goat anti-mouse KLF4 antibody (1:250, Abcam, ab129473) or without it for the negative control. The following day, the cells were incubated with a rabbit IgG biotinylated antibody (1:200; PK-4001, Vector Laboratories) for 1 h at room temperature, followed by two washes with PBS for 5 min and incubation with VECTASTAIN^®^ ABC-HRP Kit peroxidase solution. Colorimetric reactions were performed by adding DAB (Abcam, Cambridge, UK; ab64238). The number of KLF4-stained cells was counted in 10 different fields of view using the Keyence BZ-X710 microscope with a 20× objective lens. DPCs that had a clear nuclear and perinuclear staining were considered positive and cells with weak or no staining were considered negative.

### 2.4. Gene Expression of DPCs

Total RNA from DPCs cells was isolated using an RNeasy^®^ Plus Mini Kit (QIAGEN, Hilden, Germany), following the manufacturer’s instructions. First-strand cDNA was synthesized from 250 ng of total RNA using the TOYOBO ReverTra Ace PCR RT Master Mix with gDNA Remover (Toyobo, Osaka, Japan). As a positive control, the mouse embryonic multipotent stem cell line ES-D3 (American Type Culture Collection, Manassas, VA, USA) was used since it is known to express transcription factors (*Nanog*, *Oct4*, *SOX2*, and *KLF4*) [42,43] that characterize its pluripotency. For the no reaction (RT-) control, Master Mix no RT-control was used. Polymerase chain reactions (PCRs) were performed with TB Green^®^ Premix Ex Taq™ II (Takara, Shiga, Japan) in a Dice^®^ Real Time System III thermocycler (Takara) using the primers listed in Table 1. The amplification conditions were as follows: 95 °C for 30 s, followed by 40 cycles of 95 °C for 5 s and 60 °C for 30 s. The PCR amplicons were visualized with a 3% agarose gel (Agarose 21, Nippon Gene, Tokyo, Japan) on a Gel Doc™ EZ Imager (Bio-Rad, Hercules, CA, USA).

### 2.5. REP and DPC Transplantation

**Figure 1:**
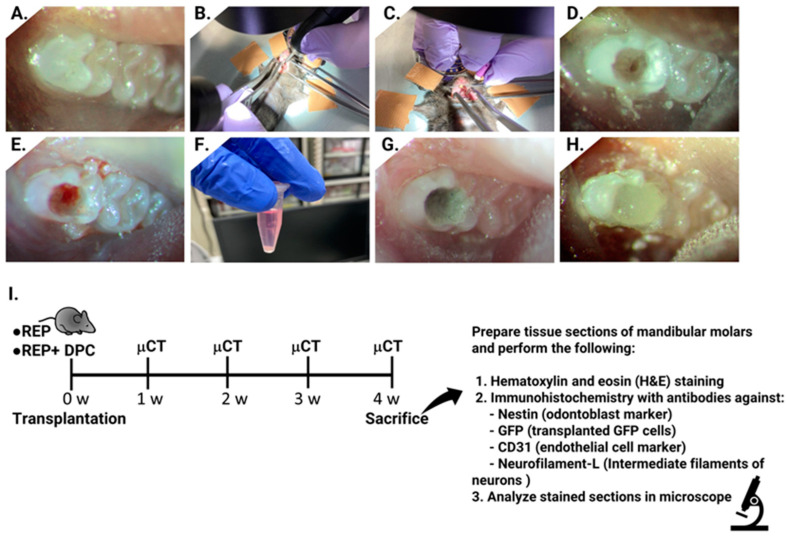
Regenerative endodontic procedure (REP) and dental pulp cell (DPC) transplantation. (**A**) C57BL/6 mouse mandibular molar. (**B**) The pulp cavity was accessed using a 1/4 round bur. (**C**) Mesial and distal root canals were instrumented with #6, #8, and #10 K files. (**D**) Root canals were irrigated with 3% ethylenediaminetetraacetic acid and 3% NaClO. (**E**) Intracanal bleeding was provoked by over-instrumentation of the canals with the K files. (**F**) DPCs (1 × 10^5^) were transplanted after intracanal bleeding (REP + DPC group) or the canals were left untreated (REP group). (**G**) Mineral trioxide aggregate was placed on the pulp cavity. (**H**) The cavity was sealed with composite resin. (**I**) Timeline of the experimental design. μCT, microcomputed tomography; GFP, green fluorescent protein; w, week.

The REP was performed on the mandibular first molars of 10-week-old C57BL/6NCr mice in a similar manner as described previously [44] with some modifications. In our previous study, radiographic analysis showed that the roots reached maturity by 8–10 weeks [45]. The mice were subject to general anesthesia via intraperitoneal injection of a mixture of medetomidine hydrochloride (0.75 mg/kg; Dormitor, Zenoaq, Fukushima, Japan), midazolam (4.0 mg/kg; Sandoz, Novartis, Basel, Switzerland), and butorphanol tartrate (5 mg/kg; Meiji Seika Pharma, Chuo City, Tokyo, Japan). The surface of the first molar and surrounding tissues were disinfected with 70% ethanol (Figure 1A). Using a stereomicroscope, cavity preparation and access to the pulp chamber were achieved using a 1/4 round bur connected to a handpiece (Figure 1B). The pulp tissue was carefully removed, and mechanical instrumentation of the canals was performed using #6, #8, and #10 K files (Figure 1C). The canals were cleaned with 3% NaClO and 3% ethylenediaminetetraacetic acid (EDTA) and the excess was dried using a cotton pellet (Figure 1D). Intracanal bleeding was induced by rotating the #6 or #8 K file with slight force until bleeding was observed (Figure 1E). Subsequently, 1 × 10^5^ DPCs were either transplanted into the bleeding canals (REP + DPC group) or the canals were left untreated (REP group) (Figure 1F). The cavity was filled with mineral trioxide aggregate (MTA) (Figure 1G) and sealed with a composite resin (CLEARFIL MAJESTY ES Flow, Tokyo, Japan) (Figure 1H). To avoid traumatic occlusion, the surface of the antagonist’s upper molar was flattened using a 1/4 round bur.

### 2.6. Micro-CT (µCT) Analysis of REP Only- and REP + DPC-Treated Molars

To examine the effectiveness of the REP treatment, in vivo µCT analyses were performed at 1, 2, 3, and 4 weeks after the treatment, as previously described [45]. Briefly, µCT images of the first mandibular molar were obtained under deep anesthesia (Cosmo Scan GX; Rigaku Corp., Tokyo, Japan) using the following parameters: 4 min, 90 kV, and 100 mA. The weekly isotropic voxel size was set at 5 mm. To detect the presence of periapical lesions, two-dimensional radiographic images in the sagittal plane were obtained from the molars with the mesial and distal roots clearly visible. The size of the periodontal space in the periapical area from 1 to 4 weeks was measured and compared using ImageJ software, 1.8.0_345 (64-bit) (National Institutes of Health, Bethesda, MD, USA), as described previously [46].

### 2.7. Analysis of the Regenerated Pulp Tissue through H&E Staining and Immunohistochemistry

Four weeks after REP or REP + DPC treatment, the mice were euthanized by carbon dioxide inhalation and the hemimandible containing the treated first molar was removed and fixed in 4% paraformaldehyde (FUJIFILM Wako, Osaka, Japan) at 4 °C for 24 h. The tissues were demineralized with 10% EDTA (FUJIFILM Wako, Osaka, Japan) for 14 days. After dehydration with a series of graded solutions of ethanol, the tissues were embedded in paraffin and 5-µm serial sagittal sections were prepared with a microtome (Leica Microsystems, Nussloch, Germany). For general histological observations, the sections were stained with hematoxylin and eosin (H&E). For immunohistochemical (IHC) staining, the sections were deparaffinized, and heat-mediated antigen retrieval was performed in a water bath at 95–100 °C for 20 min with sodium citrate buffer (pH 6.0) using chicken anti-nestin antibody (NB100-1604, Novus Biologicals, Centennial, CO, USA) and osteopontin (OPN) antibody (88742, Cell Signaling Technology, Danvers, MA, USA) and Tris-EDTA (pH 9.0) using rabbit anti-CD31 antibody (28083-1-AP, Proteintech, Rosemont, IL, USA). The sections were then incubated with 10% goat serum at room temperature for 1 h to block non-specific binding, followed by incubation overnight at 4 °C with the following primary antibodies: nestin (1:1000 dilution), OPN (1:200 dilution), CD31 (1:100 dilution), rabbit anti-neurofilament-L (1:100 dilution, C28E10, Cell Signaling Technology), rabbit anti-GFP (1:1000 dilution, 598, MBL, Tokyo, Japan), and chicken anti-GFP (1:500 dilution, ab13970, Abcam, Cambridge, UK). For the negative controls, the primary antibodies were omitted. The sections were washed with 0.05% Tween 20 in PBS three times for 5 min each and incubated at room temperature for 1 h with the following secondary antibodies purchased from Life Technologies (Carlsbad, CA, USA) at 1:200 dilutions: Alexa Fluor^®^ 594 goat anti-chicken (A11042), Alexa Fluor^®^ 594 goat anti-rabbit (A11012), Alexa Fluor^®^ 488 goat anti-rabbit (A11034), and Alexa Fluor^®^ 488 goat anti-chicken (ab150173, 1:200 dilution) purchased from Abcam. The sections were washed with 0.05% Tween 20 in PBS three times for 5 min each. Coverslips were mounted with VECTASHIELD Vibrance^®^ Antifade Mounting Medium with DAPI (H-1800, Vector Laboratories, Inc.) and slides were viewed with a KEYENCE all-in-one fluorescence microscope (BZ-X710, Keyence Corporation, Osaka, Japan). For osteocalcin IHC, the tissue sections were incubated with the primary antibody (1:1000 dilution, mOC (1-20), Takara Bio Inc., Shiga, Japan) at room temperature for 2 h after blocking endogenous peroxidase activity with 0.3% H_2_O_2_ in methanol and processed with VECTASTAIN^®^ ABC-HRP Kit peroxidase solution, following the manufacturer’s instructions. The number of CD31-positive vessel-like structures of the regenerated tissue inside the canal per tissue section was quantified using a 20× objective lens and their diameters were measured using ImageJ. The number of neurofilament-L positive nerve fibers inside the canal of the stained sections were visualized using a 40× objective lens and counted automatically using the ImageJ software, 1.8.0_345 (64-bit) after adjusting the threshold of the images from the channel that emitted the fluorescent signal of neurofilament-L immunoreactive structures.

### 2.8. Statistical Analyses

Statistical analyses were performed using GraphPad Prism software version 10.1.0 for Windows (GraphPad, Boston, MA, USA; www.graphpad.com, (accessed on 4 December 2021)). For intergroup comparisons, one-way analysis of variance followed by the Tukey–Kramer multiple comparisons test was used. For evaluations of differences between two groups, a t-test was used. Statistical significance was set at *p* < 0.05.

## 3. Results

### 3.1. Isolation and Characteristics of Mouse DPCs

**Figure 2:**
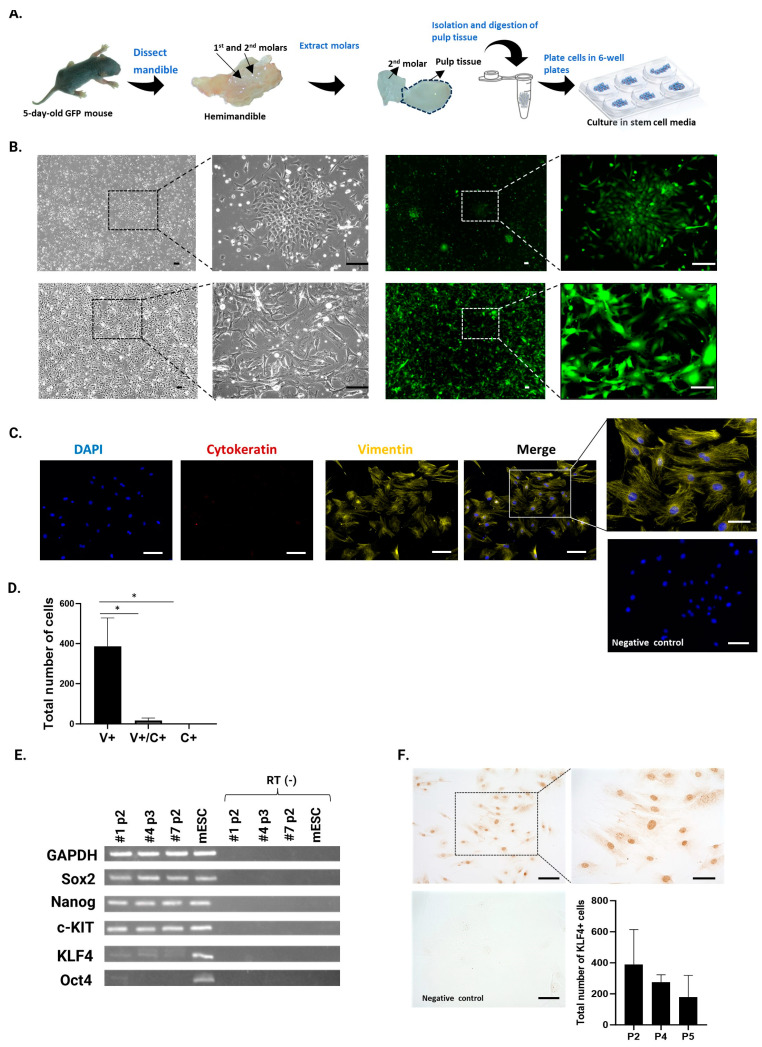
Isolation and characteristic analysis of mouse dental pulp cells (DPCs). (**A**) Dental pulp tissue was extracted from the mandibular molars of 5-day-old green fluorescent protein (GFP) transgenic mice. The tissue was incubated in enzymatic medium and DPCs were cultured in stem cell medium at 37 °C in a CO_2_-humidified incubator. (**B**) Morphological appearance and GFP expression of cultured DPCs after 1 day of culture (upper panels) and after the first passage (lower panels). Left panels are phase-contrast images (low magnification) and right panels are immunofluorescent images (high magnification). Scale bars = 100 µm. (**C**) Representative images of immunofluorescent staining of vimentin and cytokeratin in DPCs at 3rd passage. Scale bars = 100 µm (including the negative control) and 50 µm in the magnified area of the merged image. (**D**) Total number of vimentin+ (V+), vimentin+/cytokeratin+ (V+/C+), and cytokeratin+ (C+) cells quantified from 10 fields of vision with a 20× objective lens. Data are presented as means ± standard error of three different experiments using DPCs at passage 3. * *p* < 0.05. (**E**) Expression of stem cell marker genes detected by gel electrophoresis from three different samples of DPCs at the second and third passages. Mouse embryonic stem cells (mESCs) were used as a positive control. In the *Oct4* row, the order for samples #1 and #4 is reversed. (**F**) Representative images of immunohistochemical staining of KLF4 in DPCs at the fourth passage. Scale bars = 100 µm and 50 µm in the magnified panel on the right and negative control. Quantification of KLF4-positive cells in DPCs from 10 fields of vision at high magnification from two independent experiments per passage time. Data are presented as means ± standard error of the mean.

Figure 2A shows a schematic representation of the isolation of DPCs. The DPCs had a polygonal and spindle-shaped morphology, expressed GFP, and were highly proliferative, with colony formation observed after 1 day of culture. The morphology and GFP expression of the cells remained unchanged after subsequent passages (Figure 2B). To confirm that mesenchymal cells were isolated from the dental pulp tissue, vimentin (a mesenchymal cell marker) and wide-spectrum cytokeratin (an epithelial cell marker) antibody staining was performed. The results revealed that the cells were uniformly stained for vimentin and a small number of cells co-expressed cytokeratin, while there were no cells expressing only cytokeratin. (Figure 2C,D). This confirmed that the cultured DPCs had a predominantly mesenchymal phenotype. To examine whether the cultured DPCs contained stem cells, *Sox2*, *Nanog*, *c-Kit*, *Klf4*, and *Oct4* gene expression was examined using reverse transcription-PCR and gel electrophoresis. The samples expressed all the analyzed stem cell markers, except for *Oct4*, which had varied expression and was faintly detected in only one sample (Figure 2E). Among these genes, *Klf4* has been shown to promote odontoblast differentiation in DPCs by upregulating *DMP1*, *DSPP*, and *ALP* gene expression in both human DPCs and mouse dental papilla cells [47,48]. Therefore, among these markers we focused on confirming the expression of KLF4 protein in our DPC culture; thus, we performed immunocytochemical staining with a KLF4 antibody and quantified the number and percentage of KLF4-expressing cells after two, four, and five passages. The results showed that KLF4 was expressed at all passages, although with weaker staining at passage 5 (Figure 2F), even though more than half of the cells still expressed KLF4 at this stage, showing 80.72% (passage 2 (P2)), 78.63% (P4), and 60.29% (P5) of positive cells, corresponding to a total number of positive cells of 390.5 (P2), 276 (P4), and 179.5 (P5) and a total number of negative cells of 93.3 (P2), 75 (P4), and 118.3 (P5). These data confirmed that KLF4 expression was relatively constant in the DPC population, demonstrating that our DPC culture contained mesenchymal stromal cells that expressed multipotency markers.

### 3.2. µCT Analysis of the Healing Response in the REP and REP + DPCs Groups

**Figure 3:**
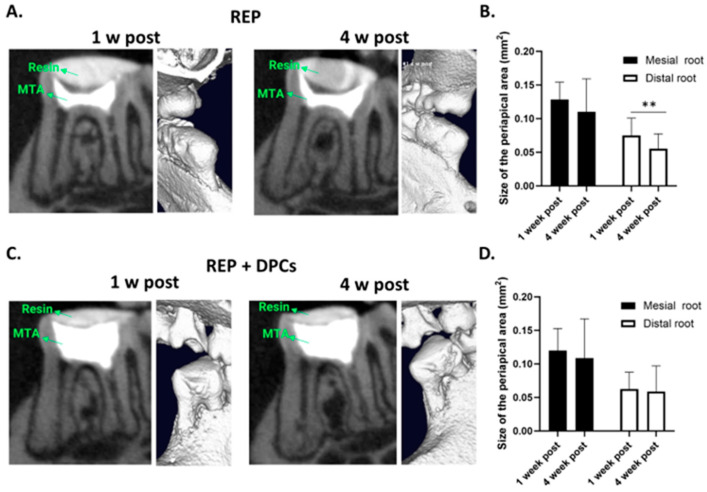
Micro-computed tomography (µCT) analysis of regenerative endodontic procedure (REP)- and REP + dental pulp cell (DPC)-treated molars. (**A**) Representative µCT images of sagittal sections and three-dimensional reconstructed images of molars from the REP (**A**) and REP + DPC (**C**) groups at 1 and 4 weeks after treatment. Measurement of the periodontal ligament (PDL) space (mm^2^) in the periapical area of the mesial and distal roots at 1 and 4 weeks in the (**B**) REP- (n = 22) and (**D**) REP + DPC-treated (n = 24) molars. Data are presented as means ± standard errors of the means. ** *p* < 0.01.

To analyze the effectiveness of the REP and/or REP + DPC treatment and the evolution of the healing response in vivo, we conducted µCT analysis of the treated molars at 1, 2, 3, and 4 weeks after treatment. The sagittal and three-dimensional reconstructed µCT images showed that the MTA applied directly into the prepared cavity remained in place and was virtually unchanged at 4 weeks after treatment. The composite resin remained adherent and sealed to the edges of the cavity, suggesting that no exogenous material was introduced during the evaluation period. The root canals showed a straight conformation, confirming the instrumentation with K files, and in most of the samples there were no signs of root resorption (Figure 3A,C). To discard the presence of periapical lesions, the periodontal ligament (PDL) space in the periapical area of the treated molars was measured at 1 and 4 weeks after treatment, showing that the size of the PDL space in both the REP and REP + DPC groups at 4 weeks after treatment in most of the samples was not increased compared to that measured at 1 week after treatment; in fact, the PDL space tended to be reduced at 4 weeks after treatment in the distal root of the REP group (Figure 3B). Overall, the data from the µCT analysis suggested that the REP and REP + DPC treatments were performed successfully and the molars had a favorable healing response.

### 3.3. Histological Analysis of the Regenerated Tissue Using H&E Staining

**Figure 4:**
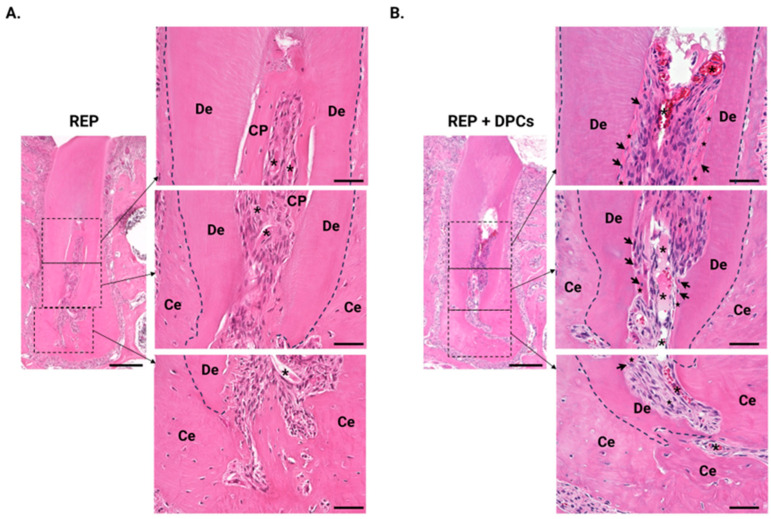
Representative images of the regenerated tissue in the regenerative endodontic procedure (REP) and REP + dental pulp cell (DPC) groups. (**A**) Regenerated pulp-like tissue in the REP group (distal root) comprising a cell-rich zone in the center of the canal with blood vessels surrounded by a thick eosin-stained cell-poor (CP) zone that resembles hard tissue. (**B**) In the REP + DPC group, the cell-rich zone is larger with abundant vessels, surrounded by a lining of eosin-stained tissue containing cells (n = 8 per group). Scale bars = 200 µm and 50 µm in the magnified panels on the right. De: dentin, CP: cell-poor zone, Ce: cementum. Asterisks: vessels, stars: regenerated dentin-like tissue; arrows: cells embedded in dentin-like tissue.

To evaluate whether tissue regeneration occurred in the mature molars after REP and/or REP + DPC treatment, molar sections were stained with H&E and analyzed under a microscope. Figure 4 shows the healing patterns in the root canals of the REP- and REP + DPC-treated molars after 4 weeks. Most of the regenerated tissue in the canals of the REP group consisted of a cell-rich zone with capillaries and vessels surrounded by a thick eosin-stained matrix cell-poor zone, characterized by cells with oval or circular nuclei similar to osteocytes or cementocytes and resembling hard tissue (Figure 4A). The canals of the molars in the REP + DPC group exhibited mixed healing patterns; some samples presented the above-mentioned healing response, whereas others had a broad cell-rich zone with abundant capillaries and vessels that transitioned seamlessly into a lining of eosin-stained tissue with cells entrapped in the matrix that had elongated nuclei (Figure 4B). Therefore, this eosin-stained zone was distinct from the bone or cementum. It should be noted that a few samples in the REP group also exhibited this healing pattern. We did not observe a clearly organized layer of cells that ran parallel to the dentin walls, similar to the physiological layer of odontoblasts, in either the REP or REP + DPC group. Overall, the confirmation of intracanal de novo tissue formation demonstrated that REP and REP + DPC treatments in mature mouse molars are achievable.

### 3.4. Characterization of the Regenerated Tissue by IHC Staining

#### 3.4.1. Detection of Odontoblast-Like Cells in the REP + DPC Group

**Figure 5:**
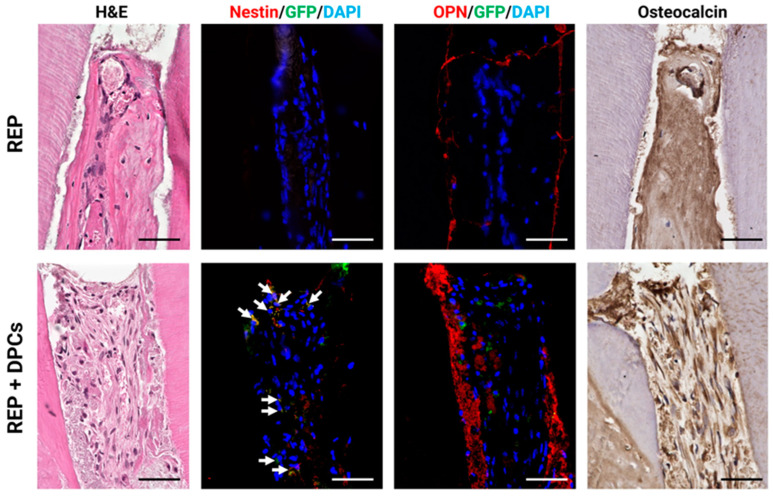
Detection of odontoblast-like cells in the regenerated dental pulp-like tissue. Representative images of hematoxylin and eosin (H&E)-stained sections and immunohistochemistry (IHC) sections stained with nestin, osteopontin (OPN), and green fluorescent protein (GFP) antibodies, as well as osteocalcin. Nestin (odontoblast marker) is not detected in regenerative endodontic procedure (REP) groups, whereas in the REP + dental pulp cell (DPC) groups, the regenerated tissue contains nestin-stained cells that co-express GFP (arrows). OPN and osteocalcin were detected in both groups, with the former being more abundant in the cellular matrix of the REP + DPC group (n = 6 per group). Scale bars = 50 µm (arrows indicate nestin and GFP co-expressing cells). 4′,6-diamidino-2-phenylindole: DAPI, Osteopontin: OPN.

The dentin–pulp matrix is a complex structure with an ensemble of cells, including odontoblasts, fibroblasts, and immune cells, which work together to maintain homeostasis. As the odontoblasts play the fundamental role of dentin production [1], it is thus desirable for the regenerated dental pulp to contain these cells to be considered a true pulp–dentin complex. To detect the presence of odontoblast-like cells in the regenerated tissue, we stained the tissues with nestin antibody (an odontoblast marker) in combination with GFP antibody to confirm whether the transplanted cells differentiated into odontoblast-like cells. Nestin-positive cells were not detected in most of the REP group, even in samples with a broad cell-rich zone (Figure 5, upper panels and Supplementary Figure S1A), with one sample showing small negligible staining in the mesial root. Unsurprisingly, GFP was also not detected since there was no transplantation of DPC cells from GFP mice in this group. A clear-cut contrast was seen in the REP + DPC samples, where nestin and GFP co-expression was detected in the cell-rich zone of the canal (Figure 5, lower panels and Supplementary Figure S1B), confirming that the cells were derived from the transplanted DPCs that differentiated into nestin-expressing odontoblast-like cells. To further confirm the presence of odontoblasts in the regenerated tissue, we proceeded to perform IHC staining with OPN and osteocalcin antibodies, both non-collagenous proteins that are synthetized by odontoblasts [49,50,51]. Interestingly, robust OPN expression was seen in the cellular matrix in the samples of the REP + DPCs groups, both near and around the GFP+ cells, while in the REP groups it was readily detected in the periphery of this cell matrix (Figure 5 and Supplementary Figure S1). Osteocalcin could be observed in both groups, with intense staining in cells from some samples in the REP + DPC groups. Overall, the expression of these markers confirmed the presence of odontoblast-like cells and indicated that DPC transplantation in combination with REP resulted in a mineralized tissue that resembled the dentin matrix.

#### 3.4.2. Vasculogenesis in the Regenerated Dental Pulp-like Tissue

**Figure 6:**
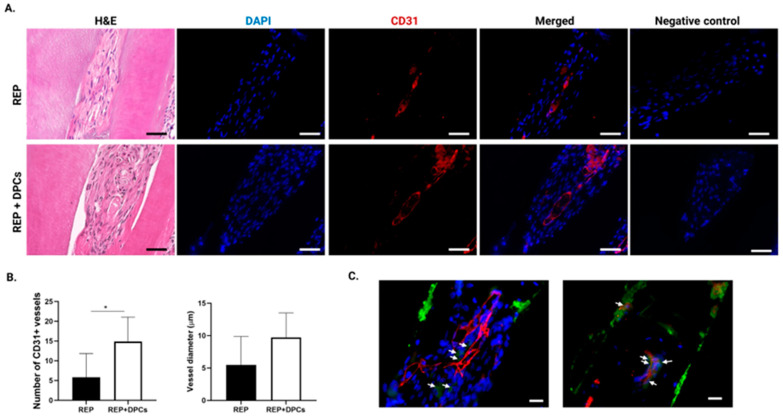
Vascularization in the regenerated dental pulp tissue. (**A**) Representative images of tissue sections from the regenerative endodontic procedure (REP) and REP + dental pulp cell (DPC) groups stained with CD31 antibody. Scale bar = 50 µm. (**B**) Quantification of vessel-like structures stained with CD31 and their diameter. Data are presented as means ± standard error of the mean (n = 6 per group). * *p* < 0.05. (**C**) Representative immunofluorescent images of green fluorescent protein (GFP)- and CD31-stained samples (n = 3, one section per sample of the REP + DPC group). Arrows indicate GFP-expressing cells in the vicinity of CD31-stained vessels. Scale bar = 20 µm. Hematoxylin and eosin: H&E, 4′,6-diamidino-2-phenylindole: DAPI, cluster of differentiation 31: CD31.

The microcirculatory system of the dental pulp is critical for homeostasis [2]. A fundamental part of REP is the formation of a blood clot in the canal that reestablishes this microcirculation, bringing about an influx of endogenous hematopoietic cells and growth factors while functioning as a scaffold for tissue regeneration [11]. To analyze whether the vascular component of the dental pulp was regenerated in the canals, we stained the molar sections with an antibody targeting the endothelial cell marker CD31. As shown in Figure 6A, CD31^+^ vessel-like structures were detected in both the REP and REP + DPC groups. This demonstrated that the REP was effective in revascularizing the canals of mature teeth, independent of DPC transplantation. To determine whether there was a difference in the degree of vascularization between the REP and REP + DPC groups, we quantified the number of vessels and measured their diameters using ImageJ software1.8.0_345 (64-bit). The results showed that molars in the REP + DPC group had a significantly greater number of CD31^+^ vessel-like structures; however, there were no differences in the diameters of these structures between the two groups (Figure 6B).

DPCs contain stem cells that can differentiate into non-mesenchymal cell lineages such as endothelial cells because of their neural crest origin [23]. Since the number of vessel-like structures was greater in the molars of the REP + DPC group, we hypothesized that the transplanted DPCs directly contributed to more prominent vasculogenesis by differentiating into endothelial cells. To this end, we performed double IHC staining using CD31 and GFP antibodies. Although we did not find vessels that clearly co-expressed CD31 and GFP, cells expressing GFP were found in close proximity to the vessels (Figure 6C). These results showed that vasculogenesis occurred in mature molars with either REP or REP + DPC treatment and to a higher degree in the latter, suggesting that transplantation of DPCs may improve vasculogenesis in REPs.

#### 3.4.3. Neurogenesis in the Regenerated Dental Pulp-like Tissue

**Figure 7:**
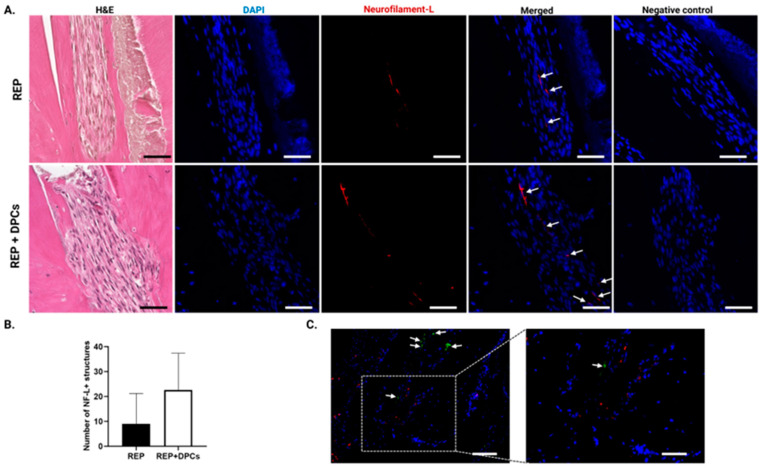
Neurogenesis in regenerated dental pulp tissue. (**A**) Representative images of tissue sections from the regenerative endodontic procedure (REP) and REP + dental pulp cell (DPC) groups stained with neurofilament-L (NF-L) antibody. Arrows indicate NF-L-stained structures. Scale bar = 50 µm. (**B**) Comparison of the number of nerve fibers between the two groups. Data are presented as means ± standard error of the mean (n = 5 per group). (**C**) Representative immunofluorescence images of neurofilament-L and green fluorescent protein (GFP) double-stained samples (n = 3). Arrows indicate GFP-expressing cells. Scale bar = 100 µm and 50 µm in the magnified panel. NF-L: Neurofilament-L.

The dental pulp is richly innervated by nerve fibers entering the pulp via the apex, which run along the long axis of the tooth together with blood vessels, providing sensory functions and tissue homeostasis [52,53,54]. To investigate if neurogenesis had occurred in the regenerated tissue, we performed IHC with neurofilament-L antibody. Neurofilament-L is a structural protein that provides support to the axonal cytoplasm and plays a role in synaptic transmission and organelle trafficking [55], which is has been used for the IHC detection of innervation in healthy and inflamed human dental pulp [56]. Neurofilament-L fibers were detected in both the REP and REP + DPC groups (Figure 7A). The REP + DPC group tended to show a higher number of fibers; however, this difference was not statistically significant (Figure 7B). The MSCs contained in the population of DPCs have the potential to differentiate into neuronal-like cells in vitro that express neurofilament-L [38]. To assess if the neurofilament-L fibers were derived from the DPCs, we performed double staining using neurofilament-L and GFP antibodies. The results did not show co-expression of neurofilament-L and GFP. In addition, in contrast to the co-expression of CD31^+^ vessel-like structures and GFP, we did not detect GFP^+^ cells near the nerve fibers. These results suggested that neurogenesis is possible in REP-treated mature teeth independent of DPC transplantation.

## 4. Discussion

In this study, we demonstrated that an REP was achievable in mature mouse molars and that the local transplantation of DPCs improved the outcomes of REPs by promoting the formation of odontoblast-like cells and enhancing vasculogenesis. In terms of methodology, the procedure itself could be regarded as successful since REP and/or REP + DPC treatment led to new tissue formation inside the canals of mature mouse molars, similar to the healing responses observed in previous preclinical studies using the mature molars of larger animals [57,58] and immature mouse molars [59]. Furthermore, the lack of periapical lesions or root resorption in almost all samples in the μCT data supports this hypothesis. Interestingly, the PDL space in the periapical region of the distal roots in the REP group tended to decrease significantly toward the end of the observation period. An initial enlargement of the PDL space is considered a normal healing response in successful cases of endodontic treatment and can be interpreted as remodeling of the periodontal tissue with gradual healing [60], especially in the case of REP treatment when bleeding is induced by widening the apex of the root. The H&E-stained sections showed mixed patterns of healing, with a higher prevalence of a cell-rich regeneration pattern in the REP + DPC group than in the REP-only group. Nevertheless, the REP + DPC group showed a tendency to have a more cellular-rich matrix, which could be attributed directly to the transplantation of DPCs that aided in forming and maintaining a tissue richer in vascularization, cellular matrix, and fibrotic tissue rather than a prominent cemento- or osteoblast-like tissue.

The term “dentin–pulp complex” is based on the intimate anatomical and physiological relationship between the dental pulp and the surrounding mineralized dentin tissue [39]. Odontoblasts in the periphery of the dental pulp are responsible for this close relationship, as these cells are involved in dentin production through their secretory projections, which become trapped in the dentin front as dentin formation occurs [61]. In cases of dental caries, odontoblasts are destroyed and a reservoir of precursor cells engages in reparative dentin formation that resembles bone [62]. However, in cases of pulpal necrosis or irreversible pulpitis, extirpation of infected tissues is necessary. Consequently, the tooth is void of any type of odontoblasts or their precursors for dentinogenesis. REP relies on intracanal bleeding, which brings progenitor cells into the canal. Lovelace et al. [8] demonstrated that stem cells were present in the canal after the induction of bleeding. Nevertheless, the scarce histological data on the outcomes of REPs in mature human teeth indicate a healing response distinct from that of the physiological dental pulp with no positive immunoreactivity to odontoblast markers and formation of a fibrous tissue with vessels and bone-like structures [12].

Another research group recently presented their findings on experimentation with REPs in immature mouse teeth [59]. Their data also demonstrated the feasibility of REPs in immature teeth from mice with open apexes, and their histological findings through H&E staining were similar to those obtained in the present work. The IHC results showed immunoreactivity to periostin, a PDL marker, in the newly formed tissue; however, no immunoreactivity to nestin was observed. Nestin is widely used as an odontoblast marker because its expression increases during tooth development and odontoblast differentiation under both physiological and pathological conditions such as caries [63,64]. However, it must be emphasized that that study was performed on immature molars with open apexes that allow for substantial blood flow compared to mature molars with closed apexes. This suggests that the influx of endogenous cells was derived from the periodontal tissue to form new cementum-like tissue and vessel-like structures, but that these cells lack odontoblast differentiation ability, suggesting that MSC transplantation is required (in this case DPCs) to form odontoblast-like cells. The DPCs used in our study contained mesenchymal stromal cells expressing stem cell-related markers, which is in agreement with a previous study [38], and the nestin expression suggests their potential for differentiation in vivo into odontoblast-like cells. OPN has a role in in odontoblast differentiation, as secretion of OPN precedes odontoblast differentiation in a transplantation model and occurs in the calcification front in parallel with the appearance of odontoblasts in murine teeth with cavity preparations [65,66]. This might imply that the tissue in the REP + DPC group is still in a state of remodeling and producing dentin at 4 weeks after treatment, as judged by the strong OPN staining observed. On the other hand, the lack of nestin expression together with the positive immunoreaction to OPN and osteocalcin in the REP group suggests that the regenerated tissue resembled bone.

Our results showed that vasculogenesis and neurogenesis were induced in both the REP and REP + DPC groups, with the former showing a higher degree of induction in the REP + DPC group. Bleeding induction serves as a biological scaffold in REPs that facilitates the influx of hematopoietic cells and growth factors to create a microenvironment suitable for vasculogenesis [11]. This was evident in both the REP and REP + DPC groups in our study. Interestingly, DPC transplantation facilitated the formation of more blood vessels. Several reports have documented the ability of DPCs to differentiate into vessels via vascular endothelial growth factor receptor 1 (VEGFR1) and heparan sulfate glycosaminoglycan as contributing factors [23,67,68]. We suspected that this higher vessel formation was due to the differentiation of DPCs into vessels. We did not detect the co-expression of GFP and CD31 in the analyzed samples; however, we detected GFP cells in the vicinity of CD31^+^ vessels. This evidence suggests that the role of the DPCs in this study is not as an orchestrator of vasculogenesis but that these cells instead have an angiogenic pericyte-like function in supporting vessel formation. This pericyte function is in agreement with previous reports that documented this function both in vivo and in vitro, which is stipulated to be VEGF2-dependent [38,40]. A recent study further shed light on the mechanics underlying the behavior of transplanted DPCs [69]; transplantation of DPC aggregates releases apoptotic vesicles that activate autophagy, thereby accelerating revascularization. This could be one of the mechanisms of action of DPCs in enhancing vasculogenesis, which warrants further analysis using the current mouse model in future studies.

Neurogenesis was detected in both the REP and REP + DCP groups; similar to the results for vascular formation, we did not detect GFP-co-expressing nerve fibers in the analyzed samples. Since the number of nerve fibers detected did not vary significantly, this effect could be independent of DPC transplantation, which is in agreement with other preclinical studies and meta-analyses of clinical studies that showed histological evidence of neurogenesis and positive responses to sensitivity tests in mature teeth treated with REPs with or without DPC transplantation [57,70].

In this study, we chose MTA as a sealing agent due to its reported biocompatibility in vitro and in clinical studies in which MTA maintains high levels of cell viability, promotes the formation of a dentin-like bridge, and ameliorates pulpal inflammation [71,72]. The direct effect of MTA in the formation of the regenerated tissue was not assessed in the current study, however the absence of nestin immunoreactivity, less OPN staining in the REP group, in addition to the instrumentation of canals which would void canals of remaining pulp tissue, suggests that MTA did not induce by itself the appearance of odontoblast-like cells. Furthermore, Komada et al. showed that REPs performed with MTA as a sealing material in immature molars did not lead to the formation of nestin immunoreactive cells [59]. While we cannot rule out that MTA influenced the differentiation of the transplanted cells, future studies can be performed to distinguish the contribution of MTA and the DPCs, for example using another material such as calcium hydroxide.

The limitations in our study also pertain to the use of DPCs which contain mesenchymal stromal cells that expressed multipotency markers. However, the percentage of cells that survived and differentiated through time was not directly evaluated. Moreover, there was a difficulty in predicting healing patterns of REP and REP + DPC which reflects the challenges behind performing REPs in mature teeth. Perhaps the period of evaluation was sufficient to analyze the new regenerated tissue, but an extended evaluation period beyond 4 weeks may bring more mature dentin matrix formation and more consistent patterns of healing throughout the different samples. These points could be examined in future studies using the current model.

## 5. Conclusions

In conclusion, this study demonstrates that an REP is achievable in mature mouse molars. Moreover, DPC transplantation improves the outcomes of REPs by enhancing vasculogenesis and promoting the formation of dentin-producing odontoblast-like cells, indicating that the hard tissue formed in the REP + DPC group was a dentin-like matrix.

## Figures and Tables

**Table 1:** Primers used in the study.

Gene	Forward primer (5′ to 3′)	Reverse primer (5′ to 3′)
*Gapdh*	TGTGTCCGTCGTGGATCTGA	TTGCTGTTGAAGTCGCAGGAG
*Sox2*	CAAAAACCGTGATGCCGACT	CGCCCTCAGGTTTTCTCTGT
*Nanog* [38]	AAGCGGTGGCAGAAAAACC	GTGCTGAGCCCTTCTGAATCA
*c-kit*	GCTCGGGCTTCTGTACAACT	AAGGCTGACTAGGGAGGAGG
*Klf4*	CCTGGCGAGTCTGACATGG	TCCTCACGCCAACGGTTAGT
*Oct4*	GAGACTTTGCAGCCTGAGGG	CTTTCATGTCCTGGGACTCCTC

## Data Availability

The data presented in this study are available upon request from the corresponding author.

## Supplementary Materials

The following supporting information can be downloaded at: https://www.mdpi.com/article/10.3390/cells13040348/s1, Figure S1: Detection of odontoblast-like cells in the regenerated dental pulp-like tissue.
