# Supplementary material for: Dental Pulp Cell Transplantation Combined with Regenerative Endodontic Procedures Promotes Dentin Matrix Formation in Mature Mouse Molars
Source: Cells. 2024 Feb 16;13(4):348. doi: 10.3390/cells13040348 (PMC10886544; doi:10.3390/cells13040348)
Supplement: Supplementary file 1 [file cells-13-00348-s001.zip › cells-2850832-supplementary.pdf]

Supplementary Figure S1

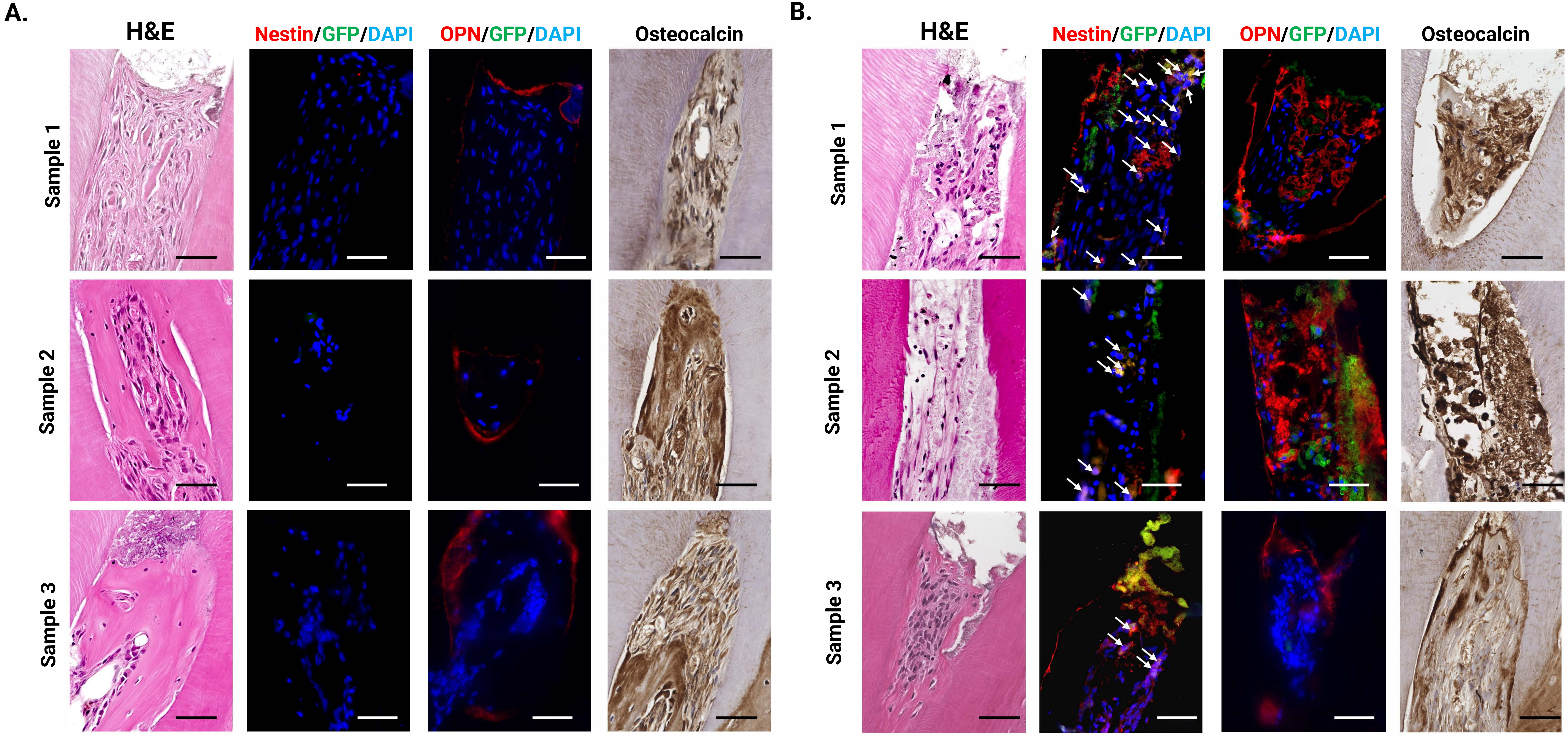

Figure S1. Detection of odontoblast-like cells in the regenerated dental pulp-like tissue. Shown are hematoxylin and eosin (H&E)-stained sections and immunohistochemistry (IHC) sections stained with nestin, osteopontin (OPN), green fluorescent protein (GFP) and osteocalcin antibodies in other samples of the regenerative endodontic procedure (REP) (A) and REP + dental pulp cell (DPC) (B) groups. Nestin (odontoblast marker) is not detected in the REP groups (A), whereas in the REP + DPC groups, the regenerated tissue contained nestin-stained cells that co-expressed GFP (B) (arrows). OPN was mainly detected in the cellular matrix of the REP + DPC group and in the periphery of the cell matrix in the REP group. Osteocalcin staining was observed in both groups. Scale bars = 50 µm. Arrows indicate nestin and GFP co-expressing cells. DAPI: 4',6-diamidino-2-phenylindole, OPN: Osteopontin.
